# The complete mitochondrial genome of the Atlantic spiny lumpsucker *Eumicrotremus spinosus* (Fabricius, 1776)

**DOI:** 10.1080/23802359.2023.2184649

**Published:** 2023-03-08

**Authors:** Likith R. Pinninti, Marius F. Maurstad, Siv N.K Hoff, Torstein Kristensen, Leslie R. Noble, Sissel Jentoft, Jorge M. O. Fernandes

**Affiliations:** a Faculty of Biosciences and Aquaculture (FBA), Nord University, Bodø, Norway; b Centre for Ecological and Evolutionary Synthesis (CEES), University of Oslo, Oslo, Norway

**Keywords:** Cyclopteridae, *E. spinosus*, Mitogenome, PacBio

## Abstract

The complete mitogenome of the Atlantic spiny lumpsucker (*Eumicrotremus spinosus)* was generated using the PacBio Sequel II HiFi sequencing platform. The mitogenome assembly has a length of 19,281 bp and contains 13 protein-coding sequences, 22 tRNA genes, 2 rRNA genes, one control region containing the D-loop (2383 bp) and a duplicate control region (1133 bp) Phylogenetic analysis using maximum likelihood revealed that *E. spinosus* is closely related to the Siberian lumpsucker (*E. asperrimus*). The mitogenome of the spiny lumpsucker will be useful in population genomics and systematic studies of Cyclopteridae, Liparidae, and Cottidae.

The Atlantic spiny lumpsucker, *Eumicrotremus spinosus* (Fabricius, 1776), is a deep-sea benthic fish native to the Arctic and coastal North Atlantic. Its morphological characteristics and particularly the presence of spiny tubercles and the ventral suction disk confirmed its taxonomic identification (Figure 1). To understand the phylogeny of Cyclopteridae family members, we sequenced the mitochondrial genome of *E. spinosus*. The specimen of *E. spinosus* was collected from the Barents Sea during an expedition as part of The Nansen Legacy project (https://arvenetternansen.com/), which was conducted by trained scientists and according to the European Union animal experimentation guidelines on the protection of animals used for scientific purposes (directive 2010/63/UE). The *E. spinosus* specimen was deposited at the University of Oslo (contact person: Sissel Jentoft; email: sissel.jentoft@ibv.uio.no) under the voucher number 787912a2-9c81-11e8-9126-8c164557e466.

**Figure 1.**
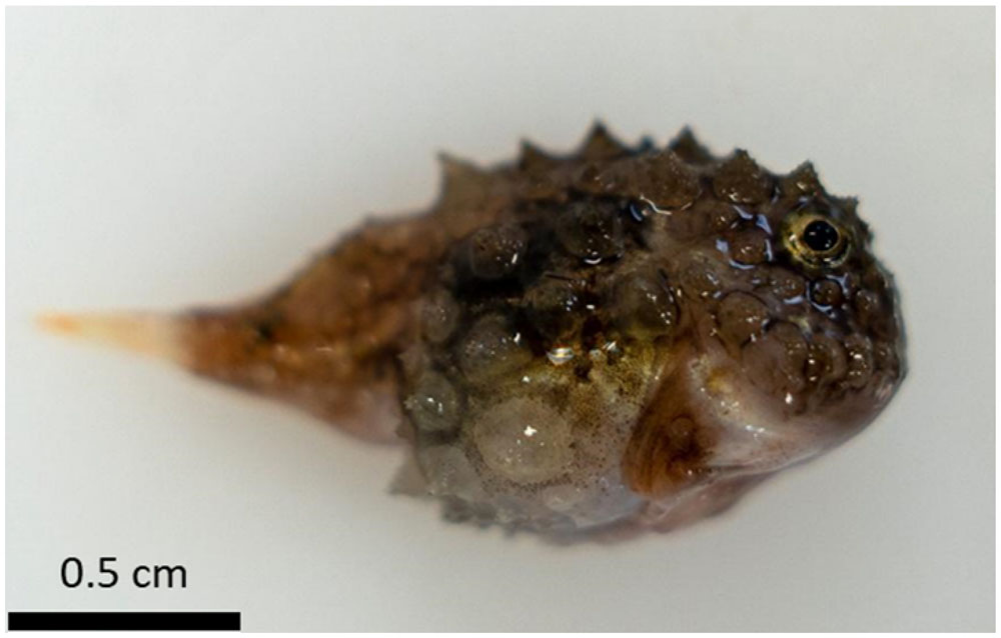
Photograph of the *E. spinosus* specimen used in the present study. The morphological traits typical of this species are spiny tubercles and a suction disk on the ventral side.

The whole fish was soaked in 96% ethanol and placed at 4 °C for two weeks; then, the ethanol was decanted before storage at −80 °C. DNA isolation and sequencing were performed by the Norwegian Sequencing Center. Genomic DNA was extracted from heart tissue using the nucleated tissue/blood protocol from the Circulomics Nanobind BIG DNA kit (Circulomics Inc.). The libraries were prepared using the Pacific Biosciences protocol for HiFi library prep using SMRTbell^®^ Express Template Prep Kit 2.0. A total of 5.77 µg DNA was sheared into 15–20 kb fragments using Megaruptor 3. After clean-up, we had ∼ 3 µg of fragmented DNA, which was used for library preparation. The final library was size selected using BluePippin with an 11 kb cutoff, resulting in ∼0.9 µg of genomic DNA. The library was sequenced on one 8 M SMRT cell on a Sequel II instrument using Sequel II Binding kit 2.2 and Sequencing chemistry v2.0. Loading was performed by adaptive loading, with a movie time of 30 h.

Mitochondrial reads were extracted from the whole genome data using MitoHiFi v2.2 (Uliano-Silva et al. 2021) with the mitogenome of *Cyclopterus lumpus* (MN122882.1) as a mapping reference. Extracted reads were assembled with Flye v2.9.1 (Kolmogorov et al. 2019) to obtain a full-length mitogenome assembly. The genome was annotated using MITOS (Bernt et al. 2013) and MitoFish annotator (Iwasaki et al. 2013). The complete *E. spinosus* mitogenome consisted of 19.2 kb (GenBank accession no. OP728784) and had a typical mitochondrial genome structure consisting of 13 protein-coding genes PCGs, 22 transfer RNA (tRNA) genes, two ribosomal RNA (rRNA) genes, and three non-coding regions (ncDNA) (Figure 2). Additionally, the control region (CR) was duplicated.

**Figure 2.**
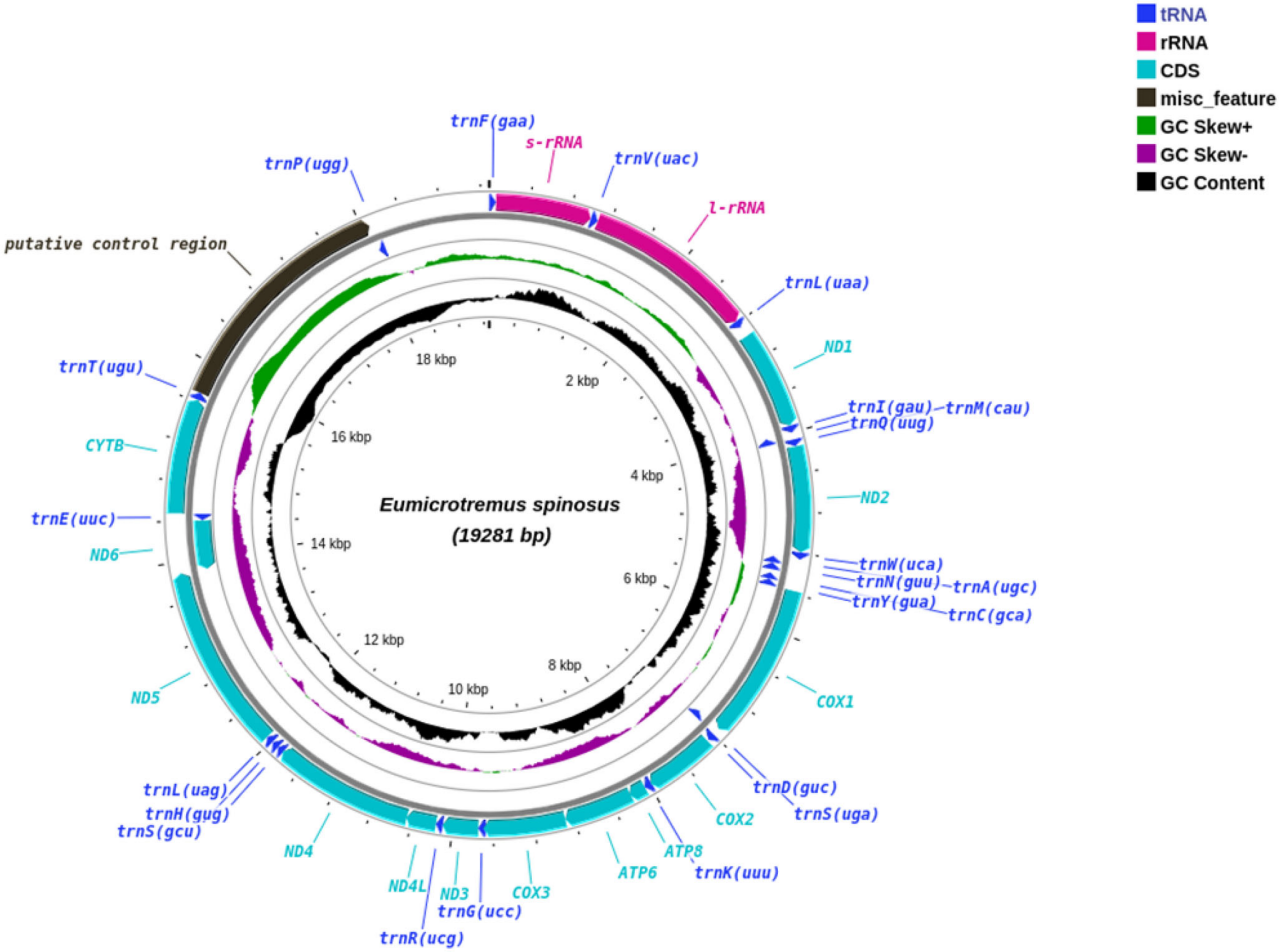
Circular plot of the Atlantic spiny lumpsucker (*E. spinosus*) mitogenome displaying both heavy (outer circle) and light (inner circle) strands. It has a typical vertebrate mitochondrial structure with 13 PCGs, two rRNA genes and 22 tRNA genes.

The *E. spinosus* mitogenome was similar to that of *C. lumpus*, which also comprises three non-coding regions, two *trnL* and two *trnS* genes (Maduna et al. 2022). In the H-strand, two CR regions, one non-coding region (the homo polymer region), 12 PCGs, and 14 tRNA genes were present. The two control regions were adjacent to each other, with the CR1 region (2383 bp) located between the *trnT* and *trnP*, and the CR2 region (1133 bp) found between the *trnP* and *trnF* genes. The presence of two CR regions is linked to concerted evolutionary events in non-coding regions (Li et al. 2015). Also, a third non-coding region (intergenic spacer) of 133 bp with a C-tract homopolymer was present between *trnL* and the *ND1* gene on the H-strand. Three non-coding regions have also been observed in other fish species (Maduna et al. 2022). The L-strand encoded the origin of replication (OL) of 43 bp, 8 tRNA genes and the *ND6* protein-coding gene. The mitogenome arrangement and structure were very similar to a typical vertebrate mitogenome, but the presence of three non-coding regions contribute to our knowledge about the diversity and organization of mitochondrial genomes.

To understand the phylogenetic relationships among the members of Cyclopteridae, Liparidae, and their closest Cottidae relatives, all their mitochondrial PCGs were aligned using MAFFT v7.471 (Katoh and Standley 2013), trimmed with trimAL (Capella-Gutierrez et al. 2009) to remove poorly aligned, divergent, and ambiguous regions, and then a maximum likelihood phylogenetic tree was generated using IQ-TREE2 v2.1.2 (Minh et al. 2020). The best-fitting evolution model was automatically selected (JC + SYM + K80) and the phylogenetic reconstruction was performed with the parameters -B 1000, -wbt and 1000 bootstrap replicates (Nguyen et al. 2015; Hoang et al. 2018). Our phylogeny followed the currently accepted taxonomic relationships between all the closely related group members of the Cyclopteridae, Liparidae, and Cottidae families (Maduna et al. 2022) with more than 94% bootstrap support for each node, as shown in Figure 3. Moreover, it confirmed that Cyclopteridae and Liparidae are sister clades and *E. spinosus* is most closely related to the Siberian lumpsucker (*E. asperrimus*). The data generated from this study will be a valuable resource for future comparative genomics and phylogenetics studies of molecular evolution and systematics in deep-sea fish.

**Figure 3.**
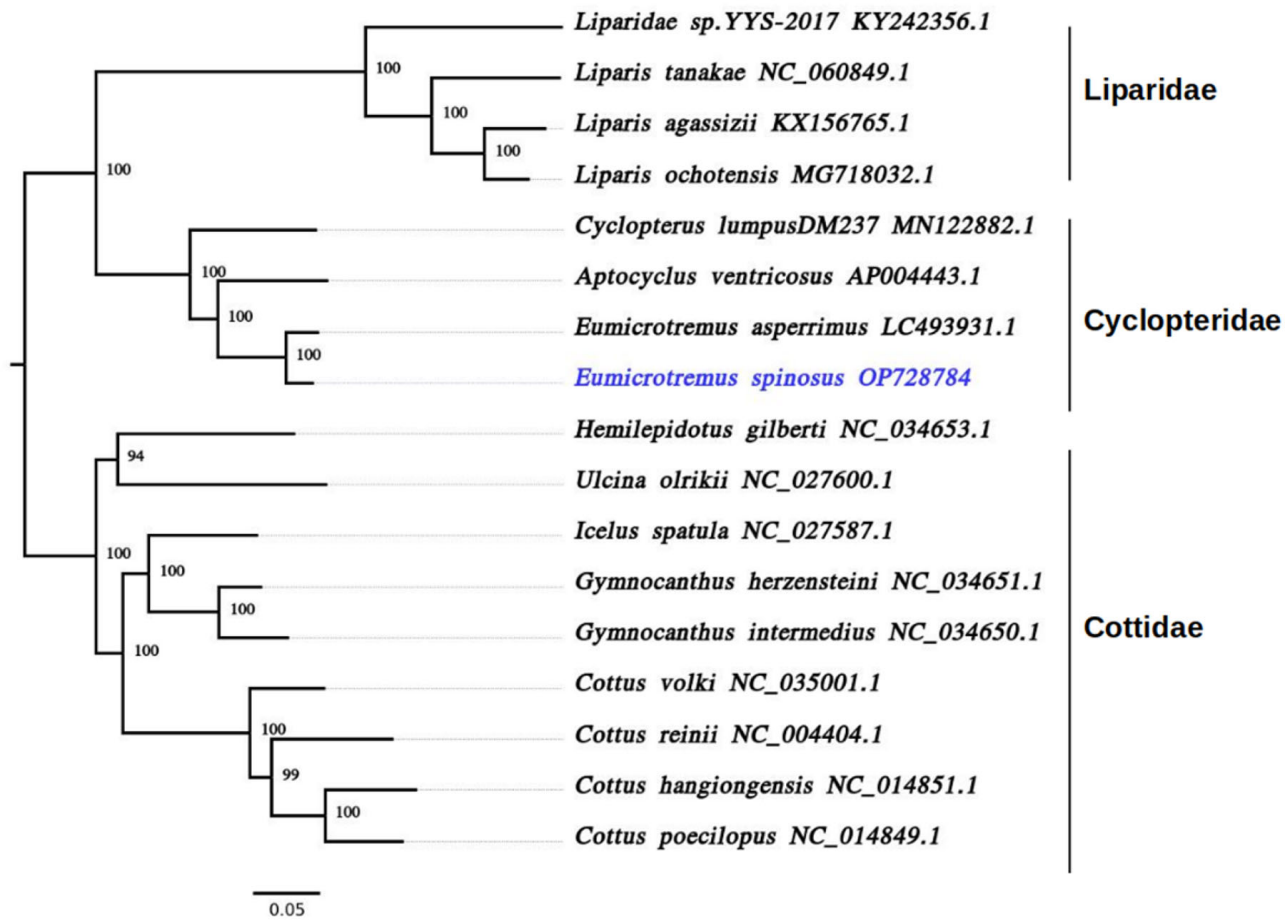
Maximum likelihood phylogeny of 17 infraorder Cottales (Teleostei: Perciformes) species based on their mitogenomes. The tree has more than 94% bootstrap support for each node. Accession numbers for each species are shown after the name of the species. The genome sequence generated in this study (OP728784) is labeled in violet and the branches are indicated in black.

## Data Availability

The mitogenome sequence data can be found in GenBank under the accession number (OP728784).

## References

[CIT0001] Bernt M, Donath A, Jühling F, Externbrink F, Florentz C, Fritzsch G, Pütz J, Middendorf M, Stadler PF. 2013. MITOS: improved de novo metazoan mitochondrial genome annotation. Mol Phylogenet Evol. 69(2):313–319. 22982435 10.1016/j.ympev.2012.08.023

[CIT0002] Capella-Gutierrez S, Silla-Martinez JM, Gabaldon T. 2009. trimAl: a tool for automated alignment trimming in large-scale phylogenetic analyses. Bioinformatics. 25(15):1972–1973. 19505945 10.1093/bioinformatics/btp348 PMC2712344

[CIT0003] Hoang DT, Chernomor O, Von Haeseler A, Minh BQ, Vinh LS. 2018. UFBoot2: improving the ultrafast bootstrap approximation. Molecular Biology and Evolution. 35(2):518–522. 29077904 10.1093/molbev/msx281 PMC5850222

[CIT0004] Iwasaki W, Fukunaga T, Isagozawa R, Yamada K, Maeda Y, Satoh TP, Sado T, Mabuchi K, Takeshima H, Miya M. 2013. MitoFish and MitoAnnotator: a mitochondrial genome database of fish with an accurate and automatic annotation pipeline. Mol Biol Evol. 30(11):2531–2540. 23955518 10.1093/molbev/mst141 PMC3808866

[CIT0005] Katoh K, Standley DM. 2013. MAFFT multiple sequence alignment software version 7: improvements in performance and usability. Mol Biol Evol. 30(4):772–780. 23329690 10.1093/molbev/mst010 PMC3603318

[CIT0006] Kolmogorov M, Yuan J, Lin Y, Pevzner PA. 2019. Assembly of long, error-prone reads using repeat graphs. Nat Biotechnol. 37(5):540–546. 30936562 10.1038/s41587-019-0072-8

[CIT0007] Li DH, Shi W, Munroe TA, Gong L, Kong XY. 2015. Concerted evolution of duplicate control regions in the mitochondria of species of the flatfish family Bothidae (Teleostei: Pleuronectiformes). PLoS ONE. 10(8):e0134580. 26237419 10.1371/journal.pone.0134580 PMC4523187

[CIT0008] Maduna SN, Vivian-Smith A, Jónsdóttir ÓDB, Imsland AK, Klütsch CF, Nyman T, Eiken HG, Hagen SB. 2022. Mitogenomics of the suborder Cottoidei (Teleostei: Perciformes): improved assemblies, mitogenome features, phylogeny, and ecological implications. Genomics. 114(2):110297. 35134501 10.1016/j.ygeno.2022.110297

[CIT0009] Minh BQ, Schmidt HA, Chernomor O, Schrempf D, Woodhams MD, Von Haeseler A, Lanfear R. 2020. IQ-TREE 2: new models and efficient methods for phylogenetic inference in the genomic era. Molecular Biology and Evolution. 37(5):1530–1534. 32011700 10.1093/molbev/msaa015 PMC7182206

[CIT0010] Nguyen LT, Schmidt HA, Von Haeseler A, Minh BQ. 2015. IQ-TREE: a fast and effective stochastic algorithm for estimating maximum-likelihood phylogenies. Mol Biol Evol. 32(1):268–274. 25371430 10.1093/molbev/msu300 PMC4271533

[CIT0011] Uliano-Silva M, Nunes JGF, Krasheninnikova K. 2021. marcelauliano/MitoHiFi: mitohifi_v2.0. https://zenodo.org/record/5205678#.Y_cvAXZBzIU

